# Neuro-Behcet disease presenting as a solitary cerebellar hemorrhagic lesion: a case report and review of the literature

**DOI:** 10.1186/s13256-016-1151-9

**Published:** 2016-12-20

**Authors:** Minju Yeo, Hye-Lim Lee, Minju Cha, Ji Seon Kim, Ho-Seong Han, Sung-Hyun Lee, Sang-Soo Lee, Dong-Ick Shin

**Affiliations:** 1 Department of Neurology, Chungbuk National University College of Medicine, Chungbuk National University Hospital, 776 1Sunhwan-ro, Seowon-ku, Cheongju-si, Chungbuk, 361-711 South Korea; 2 Department of Neurology, Yuseong Sun General Hospital, DaeJeon, 34084 South Korea

**Keywords:** Neuro-Behcet’s disease, Intracerebellar hemorrhage, Case report, Behcet’s disease

## Abstract

**Background:**

Behcet’s disease is a heterogeneous, multisystem, inflammatory disorder of unknown etiology. The classic triad of oral and genital ulcerations in conjunction with uveitis was originally described by the Turkish dermatologist Hulusi Behcet in 1937, but associated symptoms of the cardiovascular, central nervous, pulmonary, and gastrointestinal systems were later identified. In fact, Behcet’s disease with neurological involvement (neuro-Behcet’s disease) is not uncommon. Patients with neuro-Behcet’s disease typically exhibit a diverse array of symptoms, most commonly in the brainstem and diencephalic regions. Herein, we report an unusual case of neuro-Behcet’s disease in a patient who presented with a solitary cerebellar hemorrhage.

**Case presentation:**

A 39-year-old Asian woman was admitted to our hospital with complaints of a sudden speech difficulty that had manifested the same morning, and dizziness and mild vomiting experienced over the previous 3 days. Magnetic resonance images revealed target-like hemorrhagic lesions in the right hemisphere of the cerebellum. Risk factors that may result in cerebellar hemorrhage, such as high blood pressure or bleeding diathesis, were ruled out, and subsequent brain angiograms were normal.

**Conclusions:**

These findings suggest that the patient’s cerebellar hemorrhage could have been due to intracranial vasculitis in a rare, if not unique, complication of neuro-Behcet’s disease.

## Background

Behcet’s disease (BD) is a heterogeneous, multisystem, inflammatory disorder of unknown etiology. The classic triad of oral and genital ulcerations in conjunction with uveitis was originally described by the Turkish dermatologist Hulusi Behcet in 1937, but associated symptoms of the cardiovascular, central nervous, pulmonary, and gastrointestinal systems were later identified. In fact, BD with neurological involvement (neuro-BD) is not uncommon. Patients with neuro-BD typically exhibit a diverse array of symptoms, most commonly in the brainstem and diencephalic regions [1]. However, these central nervous system (CNS) abnormalities tend to resolve over time. Cerebral venous thrombosis is commonly evident on neuroimaging analyses [2]. In this report, we describe an unusual case of neuro-BD in a patient who presented with a solitary cerebellar hemorrhage.

## Case presentation

A 39-year-old Asian woman was admitted to our hospital with complaints of a sudden speech difficulty that had manifested the same morning, and dizziness and mild vomiting experienced over the previous 3 days. She had been initially diagnosed with BD in 1994, with oral and genital ulcerations and uveitis. There had been no recent head trauma.

**Fig. 1:**
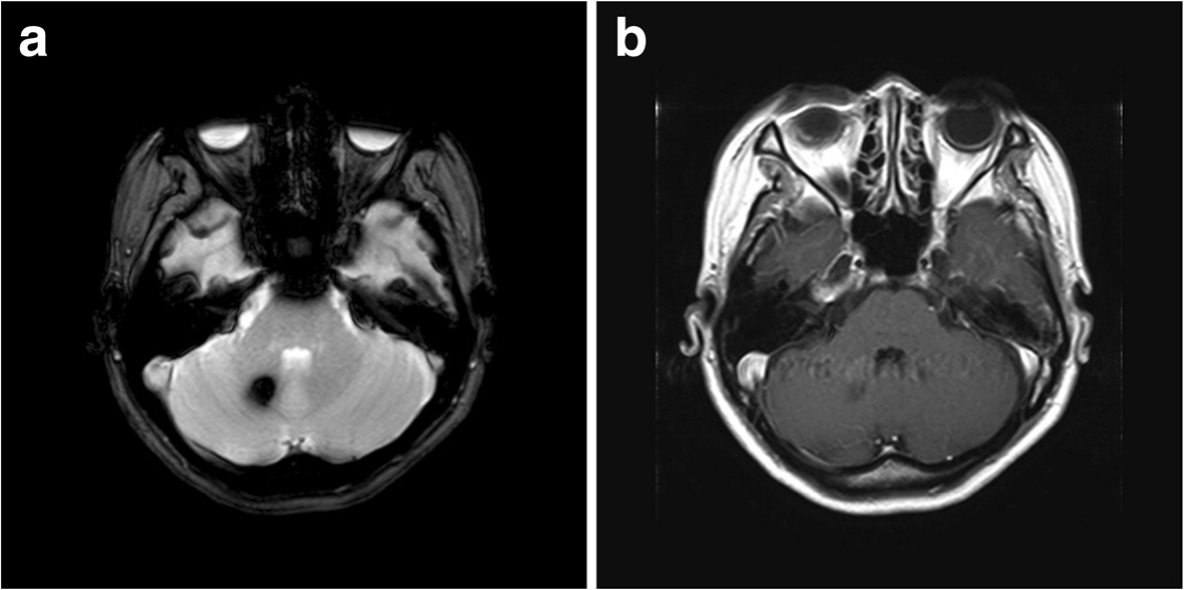
A 9.6-mm hemorrhagic lesion in the right cerebellar hemisphere. **a** T2-weighted gradient-echo cranial magnetic resonance image in the axial plane shows low signal intensity in the right cerebellar hemisphere. **b** Axial T1-weighted sequence shows no abnormally enhancing lesion following administration of intravenous gadolinium

On admission, her blood pressure was 110/70 mmHg, her pulse rate 74/min, her respiration rate 20/min, and her body temperature 36.4 °C. A physical examination was unremarkable and ophthalmoscopy did not reveal any definite lesion. A neurological examination identified dysarthria and ataxia with 2+ neck stiffness. Her erythrocyte sedimentation rate was 20 mm/h, and all laboratory findings, including indicators of liver failure, vitamin K deficiency, and disseminated intravascular coagulation, were normal. She had no relevant drug history; she had not been on antiplatelet agents or anticoagulants. An initial magnetic resonance imaging (MRI) scan of her brain was performed on the same day. Both the T1- and T2-weighted gradient-echo images revealed target-like hemorrhagic lesions in the right hemisphere of the cerebellum, along with peripheral edema and mild mass effects (Fig. 1a). Upon infusion of contrast material, the lesions exhibited subtle irregular peripheral enhancement, but no other parenchymal brain lesions were evident (Fig. 1b). Both her cerebral angiogram and her duplex carotid sonograms were normal.

We prescribed a pulse of methylprednisolone (1 g per day for 5 days); she attained near-complete recovery 2 weeks later. She was discharged and scheduled for outpatient follow-up. MRI scans taken at these visits revealed near-complete resolution of the hemorrhagic lesions; only small hemorrhagic residua were evident.

## Discussion

The earliest clinical report of neurological involvement in BD was described by Knapp in 1941, and the widely accepted term “neuro-BD” was later introduced by Cavara and D’Ermo [3]. The prevalence of CNS involvement among BD patients ranges from 4 to 49% [4–7]. Neurological symptoms most commonly manifest 3–6 years after BD onset [6–8]. However, some patients develop neuro-BD either simultaneously or prior to full-blown conventional BD [9].

According to the classification proposed by Pallis and Fudge, the neurological symptoms of BD can be divided into three categories: (1) brainstem syndrome, (2) the meningomyelitic syndrome, and (3) organic confusional syndrome [10]. In addition, vasculitis is considered to be a key feature of neuro-BD [3]; veins and arteries of any size can be affected. Venous manifestations appear to be more prevalent than those of arteries [11]. The vascular complications include cerebral venous thrombosis, and subarachnoid hemorrhages associated with intracranial aneurysms [9, 12]. However, few reports have described cerebral hemorrhages that develop in the absence of aneurysms, vascular abnormalities.

Kocer et al. described a total of 94 lesions in 65 patients with neuro-BD [1]. The most commonly affected region was the mesodiencephalic junction (disturbed in 30 patients; 46%); followed by the pontobulbar region (26 patients; 40%); the hypothalamic-thalamic region (15 patients; 23%); the basal ganglia (12 patients; 18%); the telencephalon (5 patients; 8%), the cerebellar white matter (3 patients; 5%); and the cervical cord (3 patients; 5%). The same authors described 60 patients (92%) with nonhemorrhagic lesions and 5 (8%) with hemorrhages. The nonhemorrhagic lesions exhibited prolonged T1 and T2 relaxation times. Of the hemorrhagic lesions, three were subacute, and two were hypointense on all sequences, attributable to the presence of hemorrhagic degradation products. Hemorrhagic lesions were identified in the mesodiencephalic junctions of three patients, the tectum of one patient, and the posterior perforate substance of another patient.

## Conclusions

Risk factors that may trigger cerebellar hemorrhage, including high blood pressure and bleeding diathesis, were absent in our patient, and her post-presentation brain angiograms were normal. This suggests that the cerebellar hemorrhage could have been due to intracranial vasculitis, which is a rare, if not unique, complication of neuro-BD. Hemorrhagic complications of cerebral arteritis developing subsequent to arterial inflammation and vessel wall weakening have been reported. We suggest that, in our patient, the cerebellar hemorrhage was attributable to similar venous changes, although we cannot exclude other possible causes such as venous thrombosis-associated hemorrhage, an infarct that subsequently underwent hemorrhagic transformation, or neuro-BD of uncertain etiology.

## Abbreviations

BD: Behcet’s disease
CNS: central nervous system
MRI: magnetic resonance imaging
neuro-BD: neuro-Behcet’s disease

## References

[1] Kocer N, Islak C, Siva A, Saip A, Akman C, Kantarci O, Hamuryudan V. CNS involvement in neuro-Behcet syndrome: an MR study. AJNR Am J Neuroradiol. 1999;20:1015–24. PMC7056254 10445437

[2] Wechsler B, Vidailhet M, Piette JC, Bousser MG, Dell Isola B, Blétry O, Godeau P. Cerebral venous thrombosis in Behcet’s disease: clinical study and long-term follow-up of 25 cases. Neurology. 1992;42:614–8. 10.1212/wnl.42.3.614 1549224

[3] Shahien R, Bowirrat A. Neuro-Behcet’s disease: a report of sixteen patients. Neuropsychiatr Dis Treat. 2010;6:219–25. PMC2877603 20520785

[4] Serdaroglu P. Behcet’s disease and the nervous system. J Neurol. 1998;245:197–205. 10.1007/s004150050205 9591220

[5] Farah S, Al-Shubaili A, Montaser A, Hussein JM, Malaviya AN, Mukhtar M, Al-Shayeb A, Khuraibet AJ, Khan R, Trontelj JV. Behcet’s syndrome: a report of 41 patients with emphasis on neurological manifestations. J Neurol Neurosurg Psychiatry. 1998;64:382–4. 10.1136/jnnp.64.3.382 PMC2169980 9527155

[6] Akman-Demir G, Serdaroglu P, Tasci B. Clinical patterns of neurological involvement in Behcet’s disease: evaluation of 200 patients. The Neuro-Behcet Study Group. Brain. 1999;122(Pt 11):2171–82. 10.1093/brain/122.11.2171 10545401

[7] Kidd D, Steuer A, Denman AM, Rudge P. Neurological complications in Behcet’s syndrome. Brain. 1999;122(Pt 11):2183–94. 10.1093/brain/122.11.2183 10545402

[8] Siva A, Kantarci OH, Saip S, Altintas A, Hamuryudan V, Islak C, Koçer N, Yazici H. Behcet’s disease: diagnostic and prognostic aspects of neurological involvement. J Neurol. 2001;248:95–103. 10.1007/s004150170242 11284141

[9] Al-Araji A, Kidd DP. Neuro-Behcet’s disease: epidemiology, clinical characteristics, and management. Lancet Neurol. 2009;8:192–204. 10.1016/S1474-4422(09)70015-8 19161910

[10] Pallis CA, Fudge BJ. The neurological complications of Behcet’s syndrome. AMA Arch Neurol Psychiatry. 1956;75:1–14. 10.1001/archneurpsyc.1956.02330190009001 13275156

[11] Owlia MB, Mehrpoor G. Behcet’s disease: new concepts in cardiovascular involvements and future direction for treatment. ISRN Pharmacol. 2012;2012:760484. 10.5402/2012/760484 PMC3317002 22530146

[12] Zsigmond P, Bobinski L, Bostrom S. Behcet’s disease, associated with subarachnoidal heamorrhage due to intracranial aneurysm. Acta Neurochir (Wien). 2005;147:569–71. discussion 571. 10.1007/s00701-005-0516-4 15806330

